# Network analyses of Oppositional Defiant Disorder (ODD) symptoms in children

**DOI:** 10.1186/s12888-022-03892-5

**Published:** 2022-04-13

**Authors:** Rapson Gomez, Vasileios Stavropoulos, Andre Gomez, Taylor Brown, Shaun Watson

**Affiliations:** 1 Federation University, Ballarat, Australia; 2 Victoria University, Footscray, Australia; 3 University of Athens, Athens, Greece; 4 Wings Wellness Psychology Clinic, 116 Derrimut Road, Hoppers Crossing, Victoria 3029 Australia

**Keywords:** Oppositional Defiant Disorder symptoms, Children, Parent ratings, Teacher ratings, Network analysis

## Abstract

Based on parent and teacher ratings of their children, this study used regularized partial correlation network analysis (EBIC glasso) to examine the structure of DSM-5 Oppositional Defiant Disorder (ODD) symptoms. Parent and teachers (*N* = 934) from the general community in Malaysia completed questionnaires covering DSM-5 ODD symptoms. The most central ODD symptom for parent ratings was anger, followed by argue. For teacher ratings, it was anger, followed by defy. For both parent and teacher ratings, the networks revealed at least medium effect size connections for temper and argue, defy, and argue, blames others, and annoy, and spiteful and angry. Overall, the findings were highly comparable across parent and teacher ratings, and they showed a novel understanding of the structure of the ODD symptoms. The clinical implications of the findings for assessment and treatment of ODD are discussed.

**Supplementary Information:**

The online version contains supplementary material available at 10.1186/s12888-022-03892-5.

Oppositional Defiant Disorder (ODD) is a common disorder [1, 2], and refers to a recurrent pattern of negativistic, defiant, disobedient, and hostile behavior toward authority figures that persists for at least six months [2]. Recently, the network model was proposed for understanding psychological disorders [3]. In this framework, the symptoms of a disorder are understood as a causal system, interacting with each other in meaningful ways, resulting in the disorder [3]. Although there is some network data for ODD symptoms, it is argued that there are major gaps and limitations in this area of research. In view of this, the current study used network analysis [4], controlling for these limitations, to examine the network structure of DSM-5 ODD symptoms, based on parent and teacher ratings.

## Conceptualization and latent variable models of DSM-5 ODD symptoms

For diagnosis of ODD, DSM-5 has the same eight symptoms as in DSM-IV/DSM-IV-TR [1, 2], but they are placed into three symptom groups: anger/irritable (comprising symptoms of temper tantrums, anger, and touchiness), vindictiveness (comprising the symptom for spiteful/vindictiveness), and argumentative/defiant behavior (comprising symptoms of arguing with adults, purposefully annoying others, disobedience, and blaming others for one’s own mistakes). Nevertheless, as the symptoms are the same in DSM-IV/DSM-IV-TR and DSM-5, the findings based on DSM-IV/DSM-IV-TR ODD symptoms are directly applicable to DSM-5 ODD symptoms.

The three ODD groups in the DSM-5 hint at the possibility that ODD might be multidimensional. To date, the structure of DSM-5 ODD symptoms has been examined extensively using the independent cluster confirmatory factor analysis (ICM-CFA) approach. This approach involves a priori model in which items load only onto the designated factors and have zero loadings on all the other non-designated factors. Supplementary Table S1 shows the factors and the symptom compositions of the major ODD factors in CFA models proposed in the literature. As shown in the table, both two- [5, 6] and three-factor [7, 8] models have been proposed. Reviewing these models, Gomez et al. [9] concluded that while there is some agreement for distinct factors for irritable/negative affect and headstrong/spiteful (or oppositional), there is a lack of agreement on the best factor structure for ODD, and the composition of the symptoms in the comparable primary factors in the different models.

A study by Evans et al. [10] that involved a total of 32 studies (34 models) provided details of the how often the eight ODD symptoms have been identified with three different dimensions that have emerged as core symptoms groups (irritable, defiant, and hurtful). Their evaluation of these past studies suggested touchy and anger have always loaded together on the irritability dimension. Temper has mainly loaded on this factor, and also less often on defiant dimension. The other symptoms that have loaded with high frequencies on the defiant dimension have been annoys, blames, argues, and defies. These symptoms have never loaded on the irritability dimension. Annoy and blames others have also loaded with lower frequencies on the hurtful dimension. Another symptom that has loaded on this dimension is spiteful that has also loaded about as many times on both the irritability and defiant dimensions. Thus, relative to the other ODD symptoms, the symptoms for spiteful, annoy and blames others have in various studies been grouped with different symptom groups. Thus, these symptoms can be seen as the main symptoms responsible for the inconsistencies in past CFA studies.

## Novel network model for ODD

CFA is a latent variable model. A latent variable model provides a reflective view of psychopathology. As applied to a psychological disorder, this means that there is a latent (unobservable) construct (which is the disorder in question) that causes a range of observable responses (that are the symptoms of the disorder). Seen in the context of ODD, the reflective view suggests that the ODD symptoms are responses arising from a latent ODD construct. This means that the ODD symptoms are interchangeable and equally reflective of ODD. Also, the ODD symptoms are considered to have nothing in common after controlling for the latent construct (an assumption referred to as local independence).

Although the latent variable model (like that captured in a CFA) is currently the most dominant approach for understanding psychopathologies, a newly developed perspective, called the network approach, has a different view of psychopathologies. As noted by Armour et al. [11], the idea that symptoms do not interact with each other causally (as assumed in latent variable models) is highly implausible. In the network framework, symptoms are understood as a causal system, interacting with each other in meaningful ways, resulting in the disorder [3].

A network model can be tested empirically using ‘network analysis’ [3, 12]. Network analysis is an exploratory approach that provides visual and quantitative information about symptoms that are “core” or “central” (important) to the overall network of symptoms, and the strength of connections between symptoms [3, 13]. In network analysis, one creates a network, preferably based on partial correlations between variables. As noted by Epskamp and others [14, 15], such a network can identify unique interactions between variables that cannot be identified using multiple regression analysis, and when the network analysis is exploratory it is advantageous over structural equation modelling (SEM), because there are no equivalent undirected models possible in SEM. More details of network analysis are provided in the methodology section.

## Empirical research on ODD networks

To date, there have been at least three studies that have examined the network structure of ODD symptoms [16–18]. Smith et al. [18] examined the structure of DSM-5 ODD symptoms in a mixed (those with and without ODD) group of preschool children, using zero order correlations for the network. The findings showed that the symptoms of angry, annoy, and argue were central in the network, with angry being most central. Preszler and Burns [17] examined the network structure of DSM-5 ODD symptoms together with Attention Deficit/Hyperactivity Disorder [ADHD; 2] symptoms in a group of primary school-aged children from the general community, based on mother and father ratings, using a partial correlation procedure for the network. The network found symptom clusters for ODD, and the ADHD dimensions of inattention (IA) and hyperactivity/impulsivity (HI). For the ODD symptoms, the symptom of refuse was most central, and the symptom of spiteful was least central. Also, the stronger connections were between argue and temper, and annoy and spiteful. Hukkelberg and Ogden [16] examined the network structure for ten ODD symptoms [extracted from the Antisocial Behavior Scale of the Home and Community Social Behavior Scales [19]; for parent ratings of primary-school aged children. The study used a partial correlation procedure. Although the ten ODD items were selected to cover the ODD factors of irritable, headstrong, and hurtful, it did not have a spiteful symptom, and it included symptoms not listed as ODD symptoms in the DSM-5 (i.e., ignores parents or supervisors; destroys or damages others’ property, and insults peers). Keeping this in mind, the stronger connections were between temper and easily provoked, easily provoked, and easily irritated, disrupts on-going activities, and bothers and annoys others, and bothers and annoys others and insults peers. Also, bothers and annoys others and blames others were more central symptoms, and defiant to parents showed the least centrality.

## Limitations and omissions in network analysis of DSM-5 ODD symptoms

The findings from past network studies of the ODD symptoms do not allow for any clear interpretation of the network structure of the ODD symptoms. This is not surprising as the studies have included different symptoms in their network. To date, the study by Smith et al. [18] is the only one that focused exclusively on DSM-5 ODD symptoms. Even so, the findings in this study are limited. First, the study used zero-order correlation for constructing the network, which has the potential to inflate correlations, thereby resulting in difficult to interpret and misleading results [4]. Second, the study examined preschool children, and used parent ratings of the ODD symptoms. Thus, we have no data for children and adolescents, and for teacher ratings. As ODD is highly relevant to children, and as the DSM-5 views severity of ODD in terms of the presence of ODD across settings, and as teachers are useful sources of information for clinical diagnosis, it will be also useful to know the network structure of ODD symptoms for children based on teacher reports. Third, the study did not examine the accuracy and stability of the findings for centrality and edge weights. This is a limitation, as network analysis experts have recommended that a network must also be evaluated for its accuracy and stability [4]. Fourth, as all previous studies in this area have been in western countries, we do not have any network data for ODD in non-western countries. Such data could be useful to understand ODD cross-culturally. Given these limitations and omissions, there is clearly a need for more network analysis studies, applying partial correlation approaches, involving parent and teacher ratings of children in a non-Western counter (for instance, Malaysian primary school-aged children), and for the network findings to be examined for accuracy and stability.

## Clinical importance of network analysis of the ODD symptoms

Results from network analyses of the symptoms of a disorder can have important implications for theory, assessment and diagnosis, treatment, and prevention. Traditionally, the theoretical importance of a symptom is viewed in terms of its severity which is ascertained in terms of its mean score. However, in network models, centrality—which is different from the mean score—defines the importance of a symptom. Indeed, the mean levels of symptoms can change without changes in their centrality in the network [20]. Thus, different conclusions about what are core symptoms in a disorder could be arrived at when looking at symptom centrality and symptom severity [21].

In relation to treatment, as symptoms for a disorder identified as central in a network are considered most influential in producing or maintaining the disorder, intervening on these symptoms can be expected to maximize the impact of intervention. In this respect, and given its network characteristic, focusing (i.e., causal, risk, and maintenance factors) on the central symptoms could potentially have a downstream effect in improving other symptoms. As an example, if defy is found to be the central symptoms this could mean not only using behavioral techniques that are maintaining this symptom, but also other causal and risk factors, such as parenting skills and family processes associated with the symptom that could have beneficial improvement on other symptoms and ODD as a whole.

## Aims of the present study

As noted earlier, despite the novelty and noted advantages of the network approach, to the best of our knowledge, to date, no study has so far used network analysis to examine the structure of DSM-5 ODD symptoms for primary school-age children for parent and teacher ratings. Consequently, the major aim in the current study was to use network analysis, with regularized partial correlation, to examine the network structure of the eight DSM-5 ODD symptoms (temper, argue, defy, annoy, blames others, touchy/annoyed, angry/resentful, spiteful/vindictive), in a large community sample, based on parent and teacher ratings (separately). While the majority of participants in this sample can be expected to be typically developing children, the sample would also include those with elevated levels of the ODD symptoms with potential for ODD diagnosis. Consequently, the study could reveal an overall understanding of the ODD symptoms network, but not necessarily of those with the ODD diagnosis. For each respondent, we produced a network graph, displaying the topology of the symptom network. We then evaluated statistically (edge width and centrality) the symptoms most influential in the network, and the robustness and stability of the network.

## Method

### Participants, measure, and procedure

The participants in this sample were the same as those involved in previous studies that examined the prevalence of ADHD in Malaysian primary school children [22], the structure of the ODD symptoms [23], and gender invariance [24]. Since details of the participants were provided in those papers, and due to space limitation, only a brief description of participants, measures, and procedure is provided in this paper. In all, 934 parents and teachers from the State of Johor in Malaysia participated in this study. These respondents provided ratings for ODD symptoms (obtained using the ODD symptoms in the Disruptive Behavior Rating Scale [DBRS] [25]; for 436 boys and 496 girls, between 6 and 12 years of age. The children were from fourteen randomly selected schools. For the current study, the DBRS was translated into Malay (developed via forward and backward translation by experts in both languages), with some parents completing the English version, and others completing the Malay version. Respondents rated the occurrence of each symptom over the past 6-months on a 4-point scale (0 = *never or rarely*, 1 = *sometimes*, 2 = *often*, 3 = *very often*). The Cronbach’s alpha values for parent and teacher ratings for the ODD symptoms were 0.89 and 0.94 respectively. There was no significant difference for age between boys and girls, and their ethnic background and fathers’ occupational levels did not differ significantly from the general Malaysian population. Ethical approval for the study was obtained from the University of Ballarat Human Research Ethics Committee, the Federal Ministry of Education of Malaysia, and the Ministry of Education of the State of Johor, where the study was conducted. Prospective parent participants were provided a plain language statement providing the background of the study, the consent form, the DBRS, and a return envelope. Parents were also asked to provide the child's age, gender, and ethnic background, and their willingness to have the DBRS completed by their child’s class teacher. When h consent was available, the child’s teacher was requested to complete the DBRS for the child. There was a participation rate of approximately 93%.

### Statistical network analyses

A network analysis graph comprises of nodes (the variables used for the network), and s edges (the relationships between the nodes). Edge weight refers to the strength of the relationship between two nodes. For the study, edge weights were estimated using a regularized partial correlation approach, such as g-lasso [26] that shrinks small partial correlations to 0, resulting in a sparse network, and showing only the most important relationships in it.

Jeffreys' Amazing Statistics Program (JASP) version 0.14.1.0 statistical software [27] that uses the bootnet [4] and the qgraph [28] packages from R, was used in the study to conduct the network analysis. As used in y others [29–31], we applied the extended bayesian information criterion (EBIC) glasso for the network analysis to produces the optimal degree of shrinkage, setting the hyperparameter at 0.5 [14, 32]. The network shows blue and read connections between nodes that are indicative of positive and negative relations, respectively. For both, stronger relationships are shown in terms of them being thicker and denser in colored, and nodes with stronger similarities are placed closer together.

A network can also be descried statistically [33] in terms of edge weights (indicating the correlation or partial correlation between nodes) and centrality (indicating the relative importance of the individual nodes in the network). As a central symptom is one that is highly connected to other symptoms, and its activation can be expected to spread to other symptoms. Strength (also called degree), betweenness, closeness, degree, and expected influence are commonly reported indices of centrality [34]. We used degree and expected influence as measures of centrality in the study. Degree is the sum of all direct associations a given symptom exhibits with all other nodes; and it reflects the direct influence a given node has on the network. The expected influence for a node is the absolute sum of edge weights associated with it, taking into consideration negative nodes. It is therefore easier to interpret than degree [35]. Nodes with high expected influence centrality values indicate that they are more central.

A network must also be evaluated for its accuracy and stability. The accuracy of edge weights can be evaluated using bootstrap 95% non-parametric confidence intervals (CIs) [4], with narrower CIs suggest a more precise estimation of the edge [14], and therefore more accuracy. The stability of the centrality indices can be examined using case-dropping bootstrapping [14]. In brief, it examines if the order of centrality indices remains the same after re-estimating the network with less cases (or nodes), quantified in terms of correlation stability coefficient. This coefficient reflects the correlation between the original centrality indices (based on the full data) and the correlation obtained from the subset of data representing different percentages of the overall sample. Although a correlation stability coefficient of 0.7 or higher has been suggested as being the threshold, Epskamp et al. [4] have suggested that the correlation stability coefficient should be at least 0.5. The stability of the centrality indices and edge accuracy of the network were examined using the procedures just described, with 1000 bootstraps.

## Results

### Descriptive information of data

There was no missing data. Initially we examined the mean and standard deviation (*SD*) scores. The findings are presented in Supplementary Table S2. As show, the mean score for the eight symptoms for parent ratings ranged from 0.44 to 1.08. For teacher ratings, the mean score for the eight symptoms ranged from 0.38 to 0.63. The two most severely rated symptoms were temper and touchy for parent ratings, and angry and temper for teacher rating. For both respondents, the symptom with the lowest severity rating was spiteful. Inspection of the distribution of the frequencies for each symptom indicated continuous but non-normal distribution for both parent and teacher ratings. Although the mean symptom scores for parent and teacher ratings indicate relatively low levels of the ODD symptoms, all four response options for all eight symptoms for both respondents were endorsed. As the full range of responses can be inferred as reflecting the full range of the ODD spectrum, endorsement of all four response categories for an item can be interpreted as capturing the full spectrum of the ODD symptoms.

### Visualization of the ODD network

With eight symptoms or nodes, the maximum number of edges in this network was 24. Although the EBIC glasso estimation used in the analysis did not reduce the number of edges for parent ratings, it reduced it to 22 for teacher ratings.

**Fig. 1:**
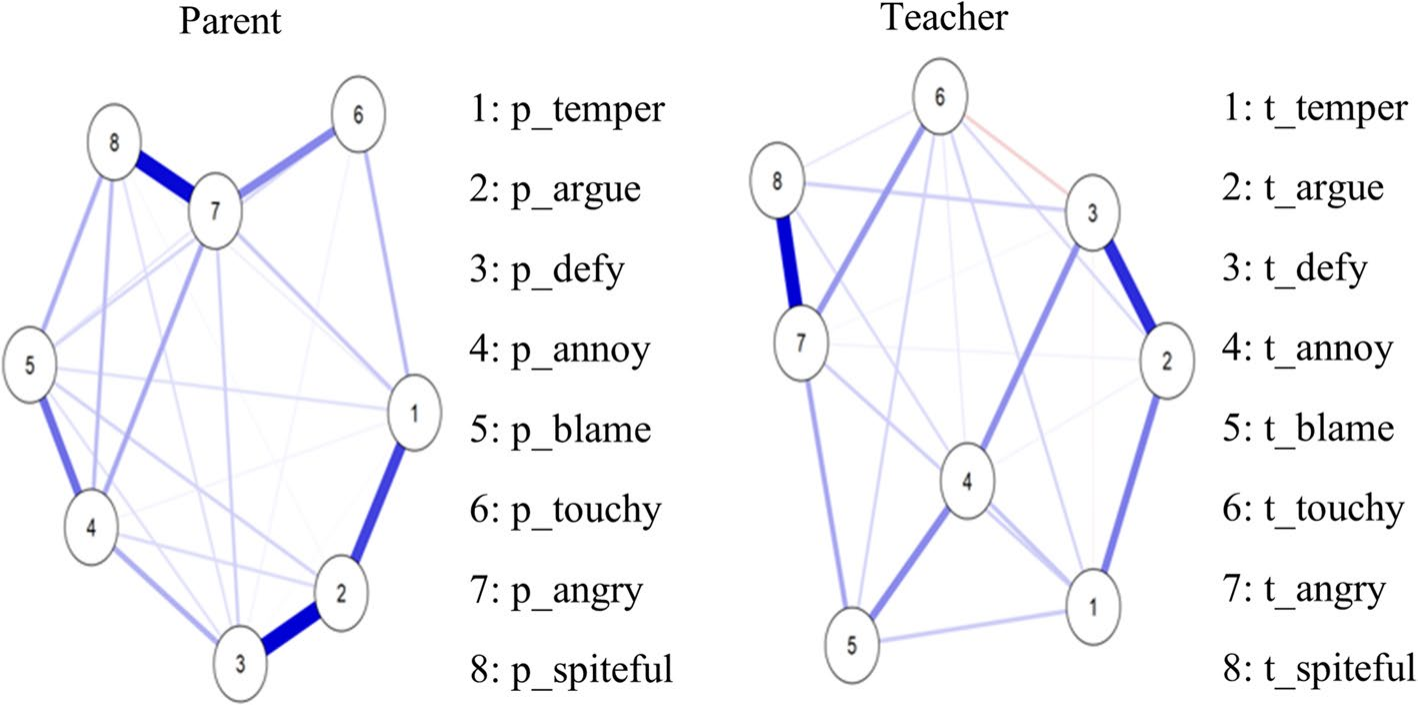
Network of the ODD symptoms based on parent and teacher ratings. Blue lines represent positive associations, and red lines negative associations. The thickness and brightness of an edge indicate the association strength. The layout is based on the Fruchterman & Reingold [36] algorithm that places the nodes with stronger and/or more connections closer together and the most central nodes into the center

Figure 1 shows a visualization of the network of the eight ODD symptoms. As shown, for parent ratings (Fig. 1, left side), the symptoms were somewhat evenly disbursed. Also, all symptoms were associated positively (blue edges) with one another. Angry (7) was placed more to the center of the network, having connections with all symptoms except for argue (2). For teacher ratings (Fig. 1, right side), all the symptoms were also relatively evenly disbursed. All symptoms were associated positively (blue edges) with one another, except for defy (3) with touchy (6) and defy (3) with temper (1). Annoy (4) was placed more to the center of this network, and it was linked to all other symptoms in the network. Also, visually, for both parent and teacher ratings, touchy (6), angry (7), and spiteful (8) were linked together relatively closely in one group in one section of the network, and temper (1), argue (2), defy (3), annoy (4) and blames others (5) were linked together relatively closely in another group in a different section of the network.

### Edge weights of ODD symptoms in the ODD network

The accuracy of the edge weights, estimated using bootstrap 95% non-parametric Cis, are shown in Supplementary Fig. S1. As shown, for both parent (left in the figure) and teacher (right in figure) ratings, the CI ranges around most of the estimated edge-weights were moderate, thereby indicating moderate precision for the edge weights. Thus, the interpretation of the edges in the networks for parent ratings and teacher ratings may be somewhat problematic.

Table 1 shows the weights matrix between the ODD nodes from the network analysis for parents (below the diagonal) and teachers (above the diagonal). For ease of interpretation, based on Cohen’s [37] effect size guidelines for correlation (*r*) values (0.1 = low, 0.3 = medium, and 0.5 = large), we interpreted *r* ≥ 0.3 (medium effect size) as important. As shown in this table, the edge weights for parent ratings (left side) meeting this cut-off score were for temper (1) and argue (2), argue (2) and defy (3), annoy (4) and blames others (5), and angry (7) and spiteful (8). Also as shown in Table 1, the edge weight findings for teacher ratings (right side) were the same as for parent ratings, with the addition that the teacher rating for annoy (4) with defy (3) also met this cut-off score of 0.30. Indeed, for both respondents, the edge weights between defy (3) and argue (2), and spiteful (8) and angry (7) were of large effect sizes. The edge weights for parent ratings showed zero values for argue (2) with touchy (6) and angry (7); and annoy (4) with touchy (6). For teacher ratings, the values were zero for temper (1) with spiteful (8); argue (2) with blames others (5) and spiteful (8); defy (3) with blames others (5); annoy (4) with angry (7); and blames others (5) with spiteful (8).

**Table 1:** Weights matrix between the ODD symptoms from the network analyses for parent and teacher ratings

# / Brief description	1	2	3	4	5	6	7	8
1 - Temper		0.35	-0.04	0.15	0.13	0.11	0.12	0.00
2 -Argue	0.39		0.57	0.03	0.00	0.10	0.03	0.00
3 - Defy	0.01	0.53		0.30	0.00	-0.12	0.02	0.12
4 - Annoy	0.02	0.07	0.16		0.31	0.06	0.00	0.09
5 - Blames others	0.06	0.09	0.06	0.30		0.11	0.23	0.00
6 - Touchy	0.14	0.00	0.03	0.00	0.08		0.27	0.06
7 - Angry	0.11	0.00	0.10	0.17	0.05	0.25		0.69
8 - Spiteful	0.03	0.02	0.07	0.13	0.16	0.00	0.51	

*Note*:Values above the diagonal are those for teachers, and values below the diagonal are those for parents

### Centrality of the ODD symptoms in the ODD network

The stability of the centrality indices (betweenness, closeness, and strength) examined using case-dropping bootstrapping are shown in Supplementary Fig. S2 (left side for parent ratings, and right side for teacher ratings). For all centrality indices, the figure shows the correlation stability (CS) coefficients from the subsets of data representing different percentages of the overall sample. Supplementary Fig. S2 shows that there was a drop (more for teacher ratings) in the correlations between the subsample estimates and the estimate from the original entire sample as the subset samples decreased from 95% of the original sample to 25% of the sample. For parent ratings, however, the correlations for the centrality indices for betweenness and strength remained above .7 for the decrease from 95% of to 25% of the sample, thereby indicating stability for the betweenness and strength centrality indices [4]. For teacher ratings, the correlations for only the centrality index for strength remained above .7 for the decrease from 95% to 25% of the sample. Strength centrality indicates how strongly a node is directly connected to other nodes. Closeness quantifies the node’s relationship to all other nodes in the network by taking into account the indirect connections from that node. Betweenness indicates how important a node is in the average pathway between other pairs of nodes. Thus, our findings for teacher ratings indicate that the order of strength centrality or how strongly a node is directly connected to other nodes is more stable than the order of closeness centrality (or the node’s relationship to all other nodes, taking into account the indirect connections from that node) and also betweenness centrality (or how important a node is in the average pathway between other pairs of nodes). Taken together, these findings indicate qualitative differences in the nature of the stability of the ODD networks for parent and teacher ratings, and also across different centrality indices for teacher ratings.

**Fig. 2:**
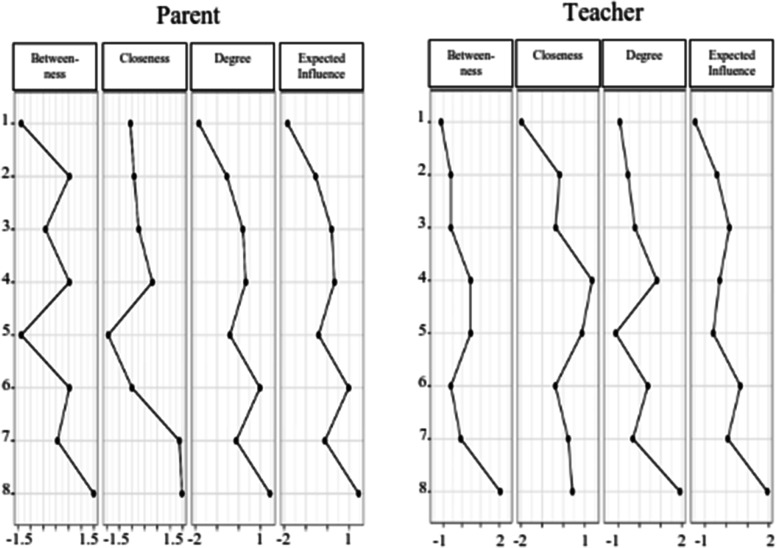
Centrality plots (betweenness, closeness, degree, and expected influence) in the Network for the Association in the Network of Each Node for Parent and teacher Ratings of the ODD Symptoms. Values shown on the x-axis are standardized *z*-scores

The standardized estimates of the centrality indices for betweenness, closeness, strength, and expected influence are presented in Table 2. To ease interpretation, plots for the centrality measures in terms of *z* scores were created, and this is displayed in Fig. 2. Both Table 2 and Fig. 2 present the centrality indices for betweenness, closeness, and degree (strength). As shown in Table 2 and Fig. 2, for the different nodes, there was notable variability in their relative values. Thus, to ensure clear interpretation of centrality, we examined strength centrality, as this index was the only one that showed stability for both parent and teacher ratings, and also because it is known to reflect reasonably precise centrality estimates for psychology networks [38]. In general, higher strength centrality values indicate more centrality.

**Table 2:** Centrality indices of ODD Symptoms from the parent and teacher ratings network analyses

	Parent			Teacher		
# / Brief description	Betweenness	Closeness	Strength	Betweenness	Closeness	Strength
1 - Temper	0.54	-0.42	-0.55	-0.60	-0.18	-0.54
2 - Argue	0.54	-0.5	0.97	-0.60	-0.38	0.40
3 - Defy	0.54	0.3	0.32	0.46	1.35	0.81
4 - Annoy	0.06	1.37	-0.12	-0.07	0.23	-0.31
5 - Blames others	-1.37	-1.44	-0.41	0.46	0.87	-1.12
6 – Touchy	-1.37	-0.56	-1.83	-1.13	-1.97	-0.93
7 – Angry	1.49	1.47	1.44	2.06	0.43	1.90
8 – Spiteful	-0.42	-0.23	0.19	-0.60	-0.36	-0.21

*Note*:Higher numbers indicate that the variable is more central to the network; highest two values are underlined within each index

As shown in Fig. 2 and Table 2, the two symptoms (in descending sequence) with the highest strength centrality values were angry (7) and argue (2) for parent ratings. For teacher ratings, they were angry (7) and defy (3). For both respondents, spiteful (8) had the lowest strength centrality value.

## Discussion

The current study is the first to use network analysis to examine the structure of the eight DSM-5 ODD symptoms [2] in a group of primary school-aged children, based on parent and teacher ratings. It examined the centrality of the ODD symptoms in the network, the edge weights for the ODD symptom pairs, and the stability and accuracy of indices for centrality and edges.

### Topology of the ODD symptom in the networks

Overall, there was reasonable level of comparability in the topology of the ODD symptom networks across parent and teacher ratings. For both parent and teacher ratings, touchy, angry, and spiteful were linked together relatively closely in one section of the network, and temper, argue, defy, annoy, and blames others were linked together relatively closely in a different section of the network. For parent ratings, angry was placed more to the center of the network, having connections with all, except the symptom for argue. For teacher ratings, annoy was placed more to the center of this network, and it was linked to all other symptoms in the network.

### ODD network findings

For parent ratings, the highest strength centrality values were angry, followed by argue. It therefore follows that for parent ratings, angry and argue may be most important ODD symptoms. For teacher ratings, the highest strength centrality values were angry, followed by defy, thereby indicating that for teacher ratings, angry and defy may be the most important ODD symptoms.

For both parent and teacher ratings, there was high connectivity between the symptoms, thereby raising the possibility that many of the ODD symptoms are closely connected with each other. For both parent and teacher ratings the edge weights with at least medium effect sizes were for temper and argue, defy and argue, blames others and annoy, and spiteful and angry. For teacher ratings only, this was also so between annoy and defy. For both parent and teacher ratings, the edge weights between defy and argue, and spiteful and angry, were of large effect sizes. Overall, therefore, there was high degree of comparability in terms of the centrality and edge weights of the symptoms across parent and teacher ratings.

### Comparison of current and past ODD network findings

Although there have been three studies that have examined networks that included the OSS symptoms [16–18] only the Smith et al. [18] study focused exclusively on DSM-5 ODD symptoms, based on parent ratings. Preszler and Burns [17] examined the network structure of DSM-5 ODD symptoms together with ADHD symptoms; and Hukkelberg and Ogden [16] examined the network structure of ten relevant ODD symptoms and not the eight DSM-5 ODD symptoms. Given this, only the network for ODD in the Smith et al. [18] study provides an appropriate comparison with the findings in the current study, and that with the findings derived from parent and not teacher ratings. In both studies, for parent ratings, all the symptoms were relatively evenly disbursed in the network. However, as already noted, in the current study, the two symptoms (in descending sequence) with the highest strength centrality values were angry and argue parent ratings. In contrast, the findings in the Smith et al. study showed that the symptoms for angry, annoys, and argues were central in the network, with anger being most central. Thus, although there were some differences, there was reasonable comparability in the findings in the current study and that reported in the Smith et al. study. This is so, despite major differences across these studies. In contrast to the current study that examined primary-school age children from the general community in an Asian country (Malaysia), Smith et al. examined the network of the ODD symptoms in a group of preschool children with and without ODD in a US sample. Additionally, the Smith et al. study used zero order correlations in the network analysis, whereas the current study used regularized partial correlation. Taking all these into consideration, it can be argued that existing findings from network analysis of ODD symptoms are robust.

### Novel clinical implications

Our findings have novel implications for theory, classification, assessment and diagnosis, and treatment and prevention, and also for explaining inconsistencies across existing models of ODD, and difference in how the irritability dimension for ODD is emphasized across the DSM-5 and the International Classification of Diseases and Related Health Problems [ICD-11] [39];. We expand on these below.

In a network, symptoms with high centrality values are considered most influential in producing or maintaining the disorder. For parent ratings, the highest strength centrality values were angry, followed by argue, and for teacher ratings, it was angry, followed by defy. Thus, it can be argued that the angry, argue, and defy symptoms are especially important for understanding and diagnosis of ODD. Individuals with serious problems related to angry and/or argue are likely to demonstrate or to be at risk for more serious ODD presentations. Thus, clinicians may wish to pay special attention to the presence of these symptoms during assessment and diagnosis of ODD. For both respondents, the spiteful symptom had the lowest centrality value, and therefore this symptom may not be critical for ODD. Notwithstanding the general commonality noted across parent and teacher ratings, they were also important differences in the centrality of the symptoms. For instances, as already mentioned, for parent ratings, the highest strength centrality symptoms were angry, followed by argue, and for teacher ratings, it was angry, followed by defy. These findings can be interpreted to indicate that the centrality of the ODD symptoms could vary in terms of respondents and/or situational effects. Our findings indicate that while argue is relatively more central than defy for the home setting, defy is relatively more central than argue for the school settings.

The theoretical importance of a symptom is traditionally viewed in terms of its severity which is ascertained in terms of its mean score. The two most severely rated symptoms were temper and touchy for parent ratings, and angry and temper for teacher ratings. However, given that in the network analysis for parent ratings, the highest two centrality symptoms were angry and argue, and for teacher ratings, it was angry and defy, different conclusions about what are core symptoms in ODD are found when looking at symptom centrality compared to symptom severity [21]. Thus, it will be useful for clinicians to also consider symptom centrality when assessing and treating children with ODD.

Since the symptoms with high centrality values are considered most influential, intervening on these symptoms could maximize the impact of an intervention, including reducing the effects of other symptoms. This, therefore, could mean that focusing intervention efforts on angry, argue, and defy symptoms rather than the other symptoms could maximize treatment effects, and also likely cascade to reduce the effects of other symptoms. Where relevant, focusing on the symptoms with high centrality values (angry, argue, and defy in the case of ODD) may also prevent the on-set and development of ODD in the context of primary prevention protocols implemented in the community.

The edge weights for parent ratings showed zero values for argue with touchy and angry; and annoy with touchy. For teacher ratings, the values were zero for temper with spiteful; argue with blames others and spiteful; defy with blames others; annoy with anger; and blames others with spiteful. The absence of a connection between two symptoms in a network implies that they are conditionally independent of each other given the other symptoms in the network. Thus, the absence of connections for these pairs of symptoms could mean that the symptoms in these relations are conditionally independent of each other. This is a novel finding and indicates that there may be a need to review the relevance of some of the DSM-5 symptoms for ODD.

For both parent and teacher ratings there was one group of closely linked symptoms comprising touchy, angry, and spiteful symptoms (group 1); and another group of closely linked symptoms comprising temper, argue, defy, annoy, and blames others symptoms (group 2). Viewed in terms of how the ODD symptoms are grouped in DSM-5, the first group for both respondents can be considered as an irritability/spiteful group; and second group can be considered as a defiant group. For parent ratings, the highest strength centrality values were angry, followed by argue. As angry and argue are symptoms in the irritability/spiteful and defiant groups, respectively, it follows that for parent ratings, angry may be the most important symptom for the irritability/spiteful group and argue may be most important symptom for the defiant group.

For teacher ratings, the highest strength centrality values were angry, followed by defy. As angry and defy are symptoms in the irritability/spiteful and defiant groups, respectively, it follows that for teacher ratings, angry may be most important symptom for the irritability/spiteful group, and defy may be most important symptom for the defiant group. Thus, there was some degree of comparability in terms of the centrality of the symptoms across parent and teacher ratings, especially for the irritability/spiteful group. Also, across the two symptom groups, the dominant connections were temper and argues for parent ratings, and spiteful and angry for teacher ratings.

As the irritability/spiteful group comprised the irritability and spiteful DSM-5 ODD symptoms, and the defiant group comprised the DSM-5 ODD defiant symptoms, the findings suggest the possibility that unlike how the ODD symptoms are grouped in DSM-5, the spiteful symptom could be placed in the angry/irritable symptom group, rather than on its own. Also, when ODD symptoms are viewed in terms the latent variable framework, a two-factor model (with latent factors for irritability/spiteful and defiant) may be most appropriate. To date ODD models with one symptom group comprising touchy, angry, and spiteful; and another symptom group comprising temper, argue, defy, annoy, and blames others; has not been proposed (see Supplementary Table S1). This model may be worthy of exploration in future studies.

Although both psychometric network modeling and latent variable modeling explain the variance–covariance structure of observed variables, they use different correlation matrices, i.e., zero-order or partial correlation versus reduced partial correlation [40, 41]. This means that we cannot directly compare the findings from CFA and network analysis studies. Statistical comparison of these models can be done using the R package Psychonetrics [42]. Notwithstanding this, our network findings do provide some possible explanation for the inconsistencies across existing CFA models of ODD. In our network analysis we used a regularized partial correlation approach that shrinks small partial correlations to 0, thereby showing only the most important relationships in it. Thus, the relations revealed in a network can be seen as the robust relationships.

As noted in the introduction, the review by Evans et al. [10] has highlighted the presence of three different ODD dimensions (i.e., irritable, defiant, and hurtful), with the symptoms for spiteful, annoy and blames others having been grouped with different ODD dimensions in different studies. Based on medium effect size for corrections (*r* ≥ 0.30), our network analysis for parent ratings, showed that spiteful (that has been grouped with either irritable, or defiant, or hurtful dimensions as reported by Evans et al.) was associated with anger (a symptom robustly associated with the irritability dimension as reported by Evans et al.); annoy (a symptom associated with the defiant and hurtful dimensions as reported by Evans et al.) was associated with blames others (a symptom associated with the defiant and hurtful dimensions as reported by Evans et al.); and blames others (a symptom associated with the defiant and hurtful dimensions as reported by Evans et al.) was associated with annoy (a symptom associated with the defiant and hurtful dimensions as reported by Evans et al.) For teacher ratings, spiteful (that has been grouped with either irritable, or defiant, or hurtful dimensions as reported by Evans et al.) was associated with angry (a symptom robustly associated with the irritability dimension as reported by Evans et al.); annoy (a symptom associated with the defiant and hurtful dimensions as reported by Evans et al.) was associated with defy and blames others (both symptoms being associated with the defiant dimensions in the study by Evans et al.); blames others (a symptom associated with the defiant and hurtful dimensions as reported by Evans et al.) was associated with annoy (a symptom associated with the defiant and hurtful dimensions as reported by Evans et al). These findings raise the possibility that spiteful, annoy and blames others may show different relations with the ODD latent factors as they all have important relations with the different ODD factors. Although our findings do not allow us the infer the underlying reason for this, it is speculated that this may be related to differences in the variance-covariance structure examined due to variation in sample characteristics.

The irritability symptom dimension of ODD has shown to be a robust predictor of anxiety and depression, and worthy of special consideration [10]. This has been dealt with different by DSM-5 and ICD-11. DSM-5 has introduced a novel mood disorder called Disruptive Mood Dysregulation Disorder (DMDD) that is effectively the irritability ODD symptoms of temper, angry, and touchy, but at a higher severity. In DSM-5, qualification for DMDD excludes the diagnosis of ODD. In ICD-11, the importance of the irritability dimension has been handled in terms of opting for a chronic irritability/anger specifier for an ODD diagnosis. As already noted, the findings in the current study for parent ratings showed that while temper and angry (symptoms for DMDD) were central symptoms for ODD, touchy (another symptom for MDDD) was not a central symptom. Instead argue and defy were central. Also, while angry was a central symptom, both temper and touchy were not central symptoms for teacher ratings. Instead, defy and blames other were central. Consequently, our findings do not support the diagnostic exclusion of ODD when DMDD is diagnosed. They are however not inconsistent with ICD-11 approach to have a chronic irritability/anger specifier for an ODD diagnosis.

### Summary of findings in the study

The most central ODD symptom for parent ratings was angry, followed by argue. For teacher ratings, it was angry, followed by defy. For both parent and teacher ratings, the networks revealed at least medium effect size connections for temper and argue, defy, and argue, blames others and annoy, and spiteful and angry. Overall, the findings were highly comparable across parent and teacher ratings, thereby attesting to their robustness. Also worthy of note is that the stability and accuracy of indices for centrality and edges were supported.

### Limitations and directions for further studies

Despite the positive value of the findings in the current study, the results in the study have to be interpreted in the light of a number of limitations. Firstly, as the study showed only moderate stability and accuracy for edge weights, the edge weight findings need to be interpreted with some caution. Secondly, network analysis assumes that mental disorders (and therefore ODD) are causal systems. However, as we used cross-sectional data in the current study, causality cannot be securely assumed. At best, we were able to eliminate spurious candidates for causal relations. Causality assessment would require longitudinal data, collected repeatedly. Further studies may wish to examine such concerns, using longitudinal network analysis. Thirdly, as we conducted the network analysis using a normative-community sample, the findings cannot be directly generalized to other samples, like specific racial and clinical groups. Fourthly, as we used parent and teacher rating measures, the findings may not be applicable to data collected via clinical interviews, or from other sources. Fifthly, as our findings are based on group-level analyses, it may not be directly applicable at the individual level. It is possible that some of the associations found in the current study may not be applicable to some individuals. Clearly, we need more network studies of the ODD symptoms, using longitudinal data, collected using multiple sources and methods and different racial and clinical groups. Individualized networks would also be beneficial for a comprehensive understanding of the ODD network. Despite these limitations, our findings do offer novel insights on the structure of ODD symptoms, their relative importance that can be used effectively for theorizing, assessing and treating ODD.

## Supplementary Information

**Additional file 1.**

**Additional file 2.**

**Additional file 3.**

## Data Availability

All data generated or analyzed during this study are included in this published article and the supplementary information file (titled data).
