# Supplementary material for: Network analyses of Oppositional Defiant Disorder (ODD) symptoms in children
Source: BMC Psychiatry. 2022 Apr 13;22:263. doi: 10.1186/s12888-022-03892-5 (PMC9009058; doi:10.1186/s12888-022-03892-5)
Supplement: Supplementary file 1 — Additional file 1. [file 12888_2022_3892_MOESM1_ESM.docx]

**Supplementary Table S1**

*Major Oppositional Defiant Disorder Models Proposed in the Literature*

| Symptoms | | Proposed Models | | | | |
| --- | --- | --- | --- | --- | --- | --- |
| # | Brief description | Rowe et al. (2010) | Burke et al. (2010) | Aebi et al. (2010) | Stringaris & Goodman (2009) | Burke et al. (2014) |
| 1 | temper | Irritable | Oppositional | Irritable | Irritable | Irritable |
| 2 | argue | Headstrong | Oppositional | Headstrong | Headstrong | Behavioral |
| 3 | defy | Headstrong | Oppositional | Headstrong | Headstrong | Behavioral |
| 4 | annoy | Headstrong | Antagonistic | Hurtful | Headstrong | Behavioral |
| 5 | blame | Headstrong | Antagonistic | Headstrong | Headstrong | Behavioral |
| 6 | touchy | Irritable | Negative Affect | Irritable | Irritable | Irritable |
| 7 | angry | Irritable | Negative Affect | Irritable | Irritable | Irritable |
| 8 | spiteful | Headstrong | Negative Affect | Hurtful | Hurtful | Behavioral |

*Note***.** The factors specified for the Burke et al. (2014) model refer to the specific factors in their bi-factor ODD model.

**Supplementary Table S2**

*Mean and Standard Deviation Scores for the ODD Symptoms in the Study*

| Symptom | |  | Parent | | Teacher | |
| --- | --- | --- | --- | --- | --- | --- |
| # | Brief description | DSM-5 grouping | Mean | SD | Mean | SD |
| 1 | temper | Angry/irritable | 1.08 | 0.77 | 0.59 | 0.68 |
| 2 | argue | Argumentative/defiant | 0.89 | 0.80 | 0.41 | 0.61 |
| 3 | defy | Argumentative/defiant | 0.73 | 0.78 | 0.38 | 0.63 |
| 4 | annoy | Argumentative/defiant | 0.63 | 0.73 | 0.46 | 0.63 |
| 5 | blame | Argumentative/defiant | 0.79 | 0.75 | 0.54 | 0.69 |
| 6 | touchy | Angry/irritable | 1.02 | 0.74 | 0.63 | 0.69 |
| 7 | angry | Angry/irritable | 0.74 | 0.75 | 0.42 | 0.63 |
| 8 | spiteful | Spiteful | 0.44 | 0.67 | 0.39 | 0.62 |
