# Supplementary material for: Network analyses of Oppositional Defiant Disorder (ODD) symptoms in children
Source: BMC Psychiatry. 2022 Apr 13;22:263. doi: 10.1186/s12888-022-03892-5 (PMC9009058; doi:10.1186/s12888-022-03892-5)
Supplement: Supplementary file 2 — Additional file 2. [file 12888_2022_3892_MOESM2_ESM.docx]

**Supplementary Figure S1**

*Edge Stability Estimate for Parent and Teacher Ratings of the ODD Symptoms - Tested Using Non-Parametric Bootstrapped Estimate*

| Parent | Teacher |
| --- | --- |
| 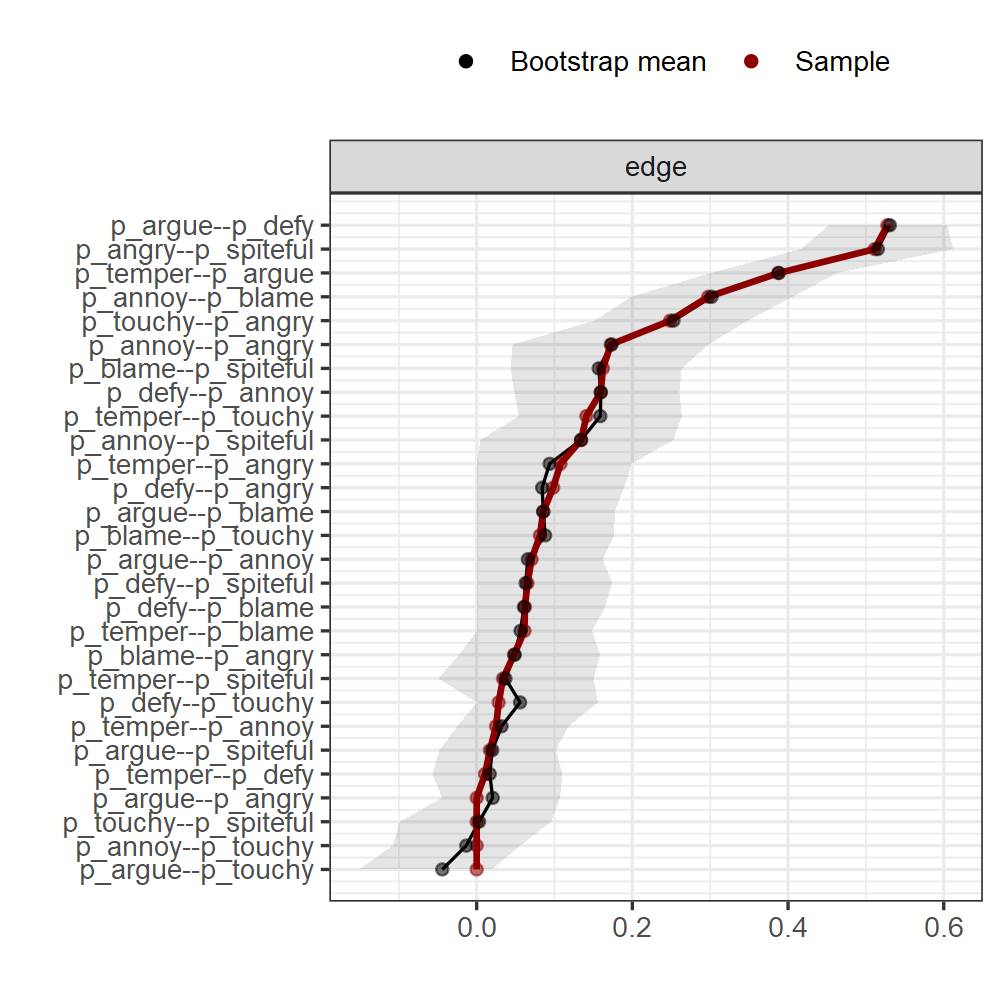 | 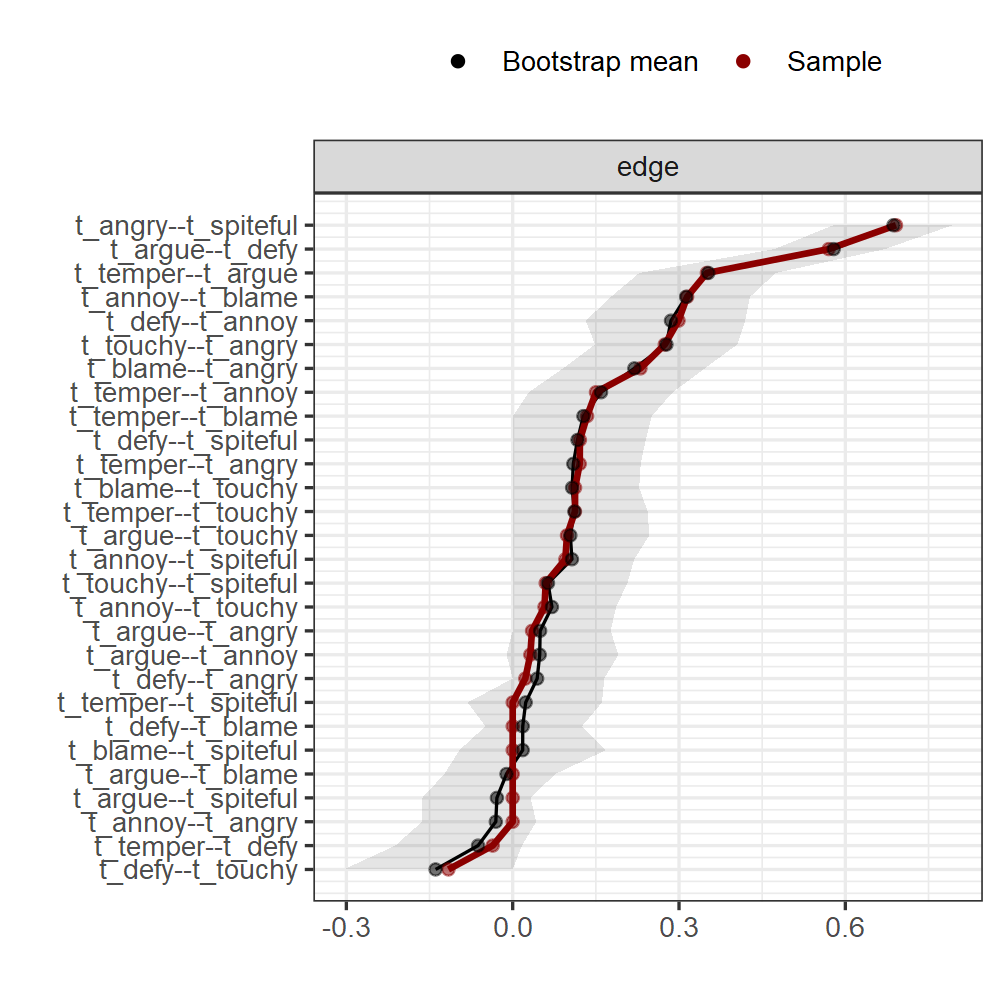 |

*Note*. The x-axis represents the edges, while every line on the y-axis represents a specific edge. The red line shows the estimate of the edge weights, and the gray bars the 95% confidence intervals for the estimates**.**

**Supplementary Figure S2**

*Stability of Central Indices for Parent and Teacher Ratings of the ODD Symptoms*

| Parent | Teacher |
| --- | --- |
| 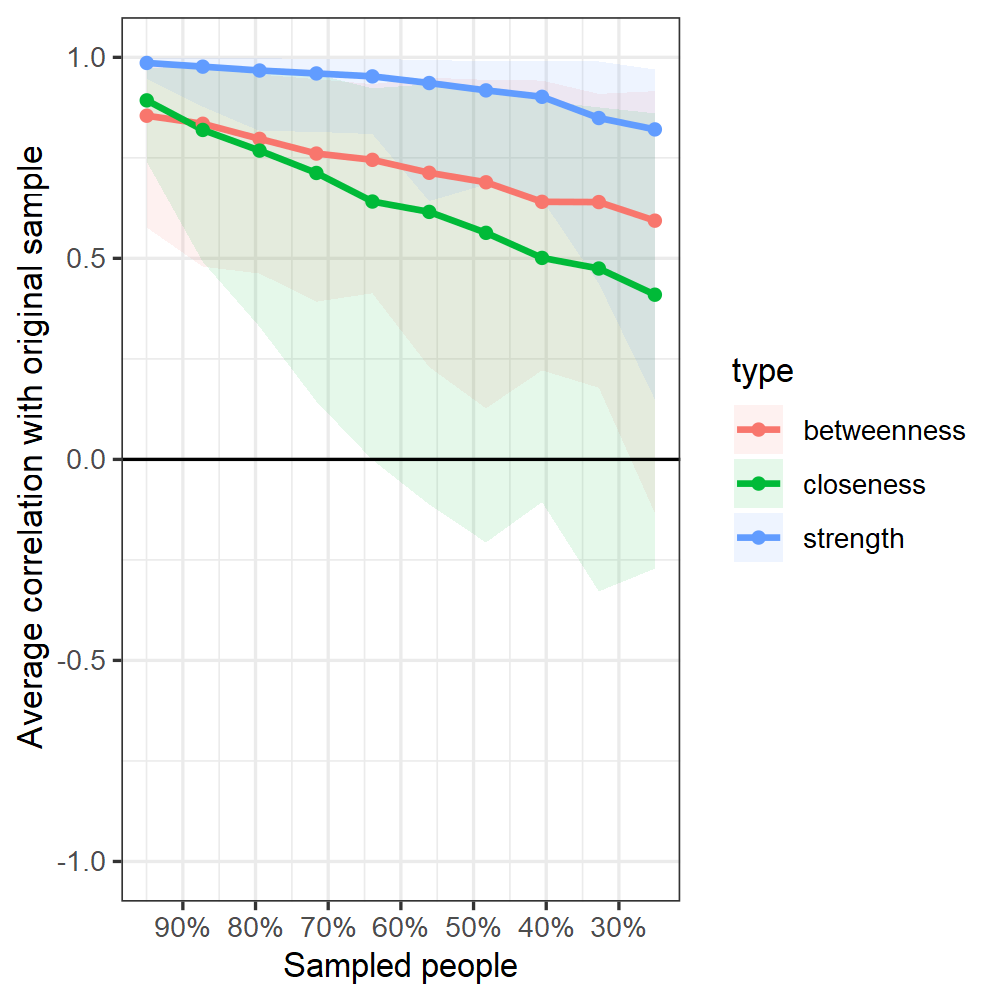 | 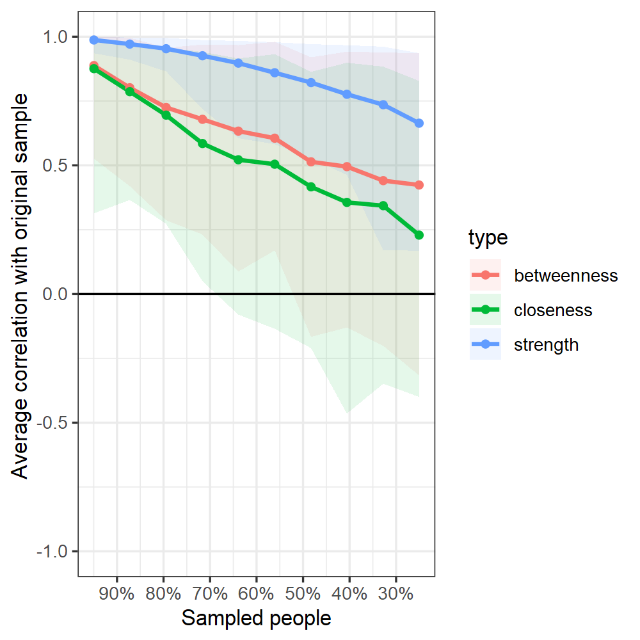 |

*Note*. The graph shows the average correlation between bootstrap centrality indices of networks sampled with node-dropping and network of the ODD symptoms.The shaded area represents the 95% confidence interval.
